# Accuracy of deformable image registration for contour propagation in adaptive lung radiotherapy

**DOI:** 10.1186/1748-717X-8-243

**Published:** 2013-10-18

**Authors:** Nicholas Hardcastle, Wouter van Elmpt, Dirk De Ruysscher, Karl Bzdusek, Wolfgang A Tomé

**Affiliations:** 1 Department of Physical Sciences, Peter MacCallum Cancer Centre, East Melbourne, VIC 3002, Australia; 2 Department of Medical Physics, University of Wisconsin-Madison, 600 Highland Ave, K4/347 CSC, Madison, WI 53792, USA; 3 Center for Medical Radiation Physics, University of Wollongong, Wollongong, NSW 2522, Australia; 4 Department of Radiation Oncology (MAASTRO), GROW – School for Oncology and Developmental Biology, Maastricht University Medical Centre, Maastricht, Netherlands; 5 Radiation Oncology, University Hospitals Leuven/ KU Leuven, Leuven, Belgium; 6 Philips Radiation Oncology Systems, 5520 Nobel Drive, 53711 Fitchburg, USA; 7 Department of Radiation Oncology, Montefiore Medical Center and Institute for Onco-Physics, Albert Einstein College of Medicine, Bronx, NY 10461, USA

**Keywords:** Deformable image registration, Adaptive radiotherapy, NSCLC, Automatic contour propagation

## Abstract

**Background:**

Deformable image registration (DIR) is an attractive method for automatic propagation of regions of interest (ROIs) in adaptive lung radiotherapy. This study investigates DIR for automatic contour propagation in adaptive Non Small Cell Lung Carcinoma patients.

**Methods:**

Pre and mid-treatment fan beam 4D-kVCT scans were taken for 17 NSCLC patients. Gross tumour volumes (GTV), nodal-GTVs, lungs, esophagus and spinal cord were delineated on all kVCT scans. ROIs were propagated from pre- to mid-treatment images using three DIR algorithms. DIR-propagated ROIs were compared with physician-drawn ROIs on the mid-treatment scan using the Dice score and the mean slicewise Hausdorff distance to agreement (MSHD). A physician scored the DIR-propagated ROIs based on clinical utility.

**Results:**

Good agreement between the DIR-propagated and physician drawn ROIs was observed for the lungs and spinal cord. Agreement was not as good for the nodal-GTVs and esophagus, due to poor soft-tissue contrast surrounding these structures. 96% of OARs and 85% of target volumes were scored as requiring no or minor adjustments.

**Conclusions:**

DIR has been shown to be a clinically useful method for automatic contour propagation in adaptive radiotherapy however thorough assessment of propagated ROIs by the treating physician is recommended.

## Background

Dose response data has shown that escalating the tumour dose can improve the probability of local tumour control [1-4] in radiotherapy for non-small cell lung cancer (NSCLC). Escalation of tumour dose without exceeding tolerance doses requires the use of highly conformal treatment techniques. Anatomical changes are often observed during radiotherapy treatment of lung cancer; both normal tissues and tumor volumes can deform over time as a response to the radiation therapy. This can impact the delivered dose when using highly conformal treatment techniques. Obtaining volumetric images during treatment fractions allows tracking of any anatomical changes for adaptive protocols, thus allowing for maintenance of treatment objectives. It has been shown that adaptive radiotherapy (ART) for shrinking lung tumours allows dose escalation and can reduce dose to normal tissues [5,6].

ART requires re-contouring of target and organ at risk (OAR) regions of interest (ROIs) for evaluation and re-planning. Currently, re-contouring of images during a radiotherapy course can be very time-intensive. Automatic propagation of target and OAR contours between two image sets is thus an attractive approach to reducing adaptive radiotherapy resource requirements [7]. DIR has been investigated extensively for automatic propagation of ROIs in 4DCT lung image sets [8-14] where the anatomy is highly correlated but subject to respiratory motion. DIR has also been investigated for automatic propagation of ROIs in adaptive radiotherapy for head and neck cancer [15-17]. However, there has been limited investigation of DIR for automatic propagation of ROIs in adaptive lung radiotherapy where significant anatomical changes due to disease progression or response to radiotherapy may be observed between images; Lu et. al. investigated automated ROI propagation between kVCT and MVCT scans in the Tomotherapy adaptive lung radiotherapy paradigm [18]. It is also acknowledged that some DIR algorithms may be more suitable for specific anatomies due to different image features and the mathematical basis of DIR algorithms. Therefore the aim of this study was to evaluate the clinical utility of three theoretically different DIR algorithms for the purpose of propagating contours from pre- to mid-treatment lung kVCT scans in the adaptive radiotherapy setting.

## Methods and materials

### DIR for contour propagation

Pre-treatment and repeat 4D respiratory correlated PET/CT scans together with a 3D intra-venous contrast-enhanced CT scan were obtained for 17 NSCLC patients. The study was approved by the Institutional Review Board. The repeat 4DCT scan was obtained during the second week of treatment, typically around the 8^th^ day after the start of radiation therapy, the details of which can be found in Van Elmpt et al. [19]. The voxel sizes for all CT scans used in this study were 0.98 mm × 0.98 mm in plane, with 3 mm slice thickness. The 50% exhale phase image was used for structure delineation in both scans. The primary tumor (GTV), lungs, esophagus and spinal cord were delineated by an expert physician on each of the pre- and mid-treatment scans. The heart was typically not contoured in the clinical workflow for these patients so was not included in this study. The nodal-GTV was delineated on 12/17 patients using FDG-PET image data for determination of lymph node involvement. The remaining 5/17 patients did not have nodal-GTVs. Pre-treatment delineation of involved lymph nodes was then performed using a contrast-enhanced CT image. Mid-treatment delineation was performed using the mid-ventilation phase (50% exhale) of a 4DCT image. DIR was performed on the mid-ventilation phase of the 4DCT, deforming the pre-treatment to the mid-treatment scans and applying the resulting deformation map to the pre-treatment scan ROIs to obtain ROIs on the mid-treatment scan. Prior to deformation, rigid registration was performed using an automated local correlation algorithm to improve the initial registration used as input to the DIR algorithms.

Three DIR algorithms were used – Demons (Fast Symmetric) [20], Salient Feature Based Registration (SFBR) [21], as implemented in a research version of the Pinnacle™ RTPs (v9.100, Philips Medical Systems, Fitchburg, WI), and Morphons [22]. The Demons algorithm was a modified version of that used in the Insight Segmentation and Registration Toolkit (ITK) [23]. The Demons algorithm uses a regular grid of forces to deform an image to a target image based on matching intensity values between two images. The optical flow equation is used to derive the displacement orientation and magnitude. The Fast Symmetric algorithm used in this study is a multi-resolution approach whereby a maximum of 200, 100, 100 and 30 iterations are run at each resolution level from 8× the image resolution to 1× the image resolution respectively. At each resolution level the standard deviation of the Gaussian smoothing was 3, 3, 0.9 and 0.7 mm for resolution levels 8 times to 1 times the image resolution. Histogram matching was performed with 64 levels and 7 match points.

The SFBR algorithm is an automated version of landmark-based registration in which sharply prominent and distinctive features are automatically located in each image. The features are found using an interest point detector algorithm and can be detected at any point in the patient. Typically 1000–2000 features are obtained. Once the features have been located, they are assigned a location determined by the position of their centroid as well as a scale. The salient feature locations are then used as anchor points to interpolate a non-rigid transformation using the Thin Plate Splines (TPS) method.

The Morphons algorithm uses quadrature phase differences to estimate deformations between two images [22,24]. Quadrature phase differences describe local structure such as edges between dark and bright areas in an image. Due to the use of quadrature phase differences this method is invariant to image intensity and weak gradients [25]. A multi-resolution implementation of Morphons was used applying 8 resolution steps with the final resolution equal to the full resolution of the CT scan with at maximum 20 iterations per resolution step, and at maximum 4 for the final grid size. Gaussian smoothing was applied between all resolution steps with a standard deviation of 1.25 times the voxel size of the resolution grid. The Morphons algorithm was implemented in Matlab (R2009a, The Mathworks, Natick, Ma) [25].

The Demons and Morphons algorithms result in a deformation vector field (DVF) at the image resolution, in the frame of reference of the second (during treatment) image. DIR was performed by deforming the pre-treatment scan to the mid-treatment scan. Propagation of the ROIs is then performed by looking up the value of the DVF in each voxel in second image and tracing it back to the pre-treatment image and obtaining the value of the binary mask of each ROI at that location. ROIs were propagated using the same technique for both the Demons and Morphons DVF. The SFBR algorithm results in a set of TPS equations that can be evaluated at any point in the image. For propagation, the source ROIs are converted to meshes and the TPS is evaluated at each vertex location in the meshes to determine the location of each mesh in the second image. No smoothing of the propagated ROIs was performed for any algorithm.

The DIR-propagated ROIs were compared with the physician-drawn ROIs on the mid-treatment scan using the Dice score and the mean slicewise Hausdorff distance (MSHD). The Dice score for two ROIs A and B was defined as 2|A∩B|/(|A|+|B|). The MSHD is the average over all slices of the largest value of the smallest distance to agreement between two ROIs on each slice [26]. The difference in the Centre of Mass (COM) position of the GTVs was also measured, as this has implications on isocenter placement in adaptive re-planning. A one-way Analysis of Variance (ANOVA) test was carried out to determine the statistical significance of any differences between the two algorithms. A value of *p* = 0.05 was used as the threshold for statistical significance.

In addition to the quantitative metric scores, the ROIs were evaluated qualitatively by an expert physician to determine the clinical utility of the algorithms for ROI propagation. A score of 1 was given to ROIs that were clinically acceptable without modification, 2 was given to ROIs that were clinically useful but required minor modification on several slices, and 3 was given to ROIs that were not clinically useful, where it would be more efficient to start the contouring from scratch.

## Results

**Figure 1:**
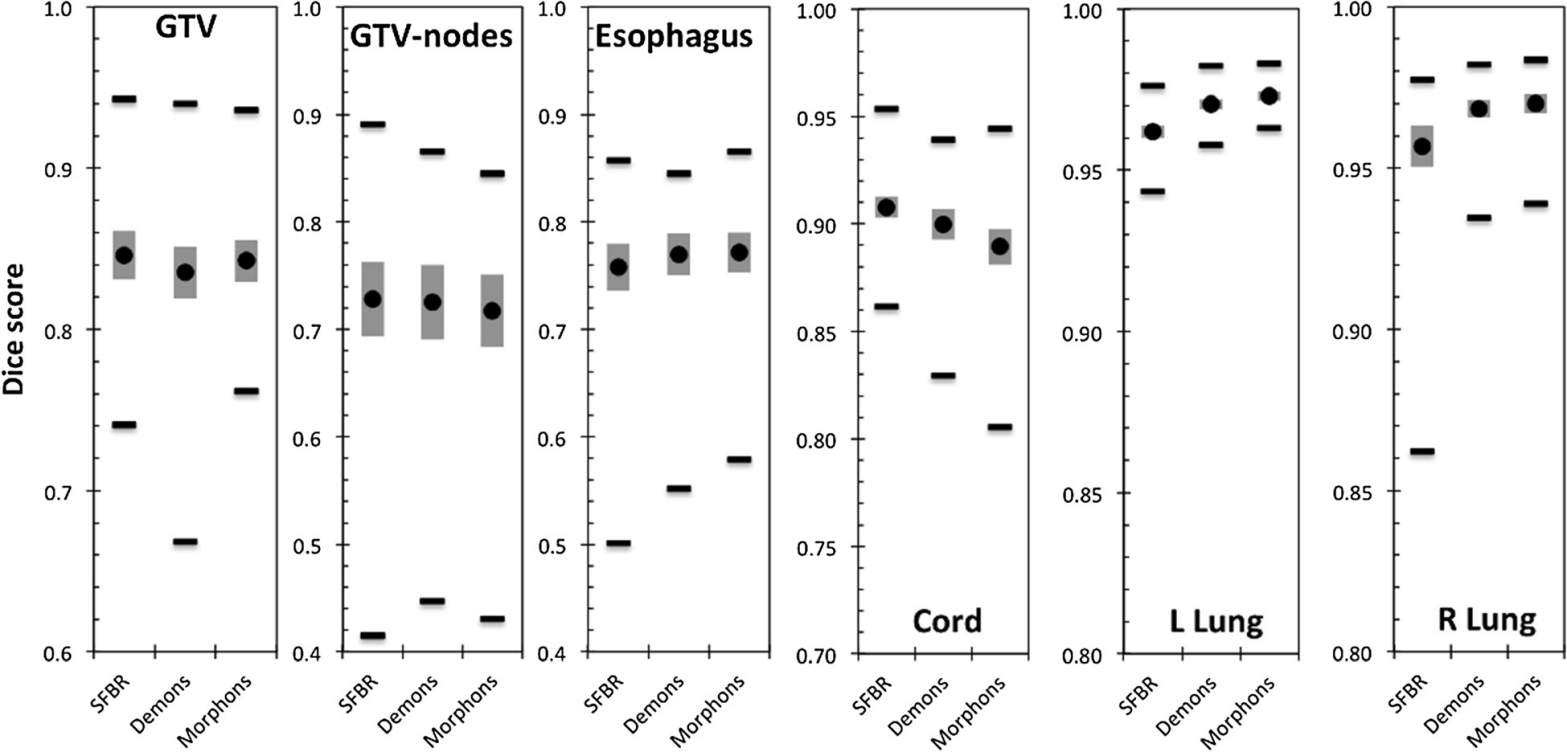
**Dice scores for the target and OARS investigated.** The closer the Dice score to 1, the better the agreement between the ROIs. The black circle is the average over all patients, the grey vertical bar is the standard error and the black horizontal lines are the ranges.

Table 1 shows the change in GTV volume between the pre- and mid-treatment images: GTV volumes reduced in 14 out of 17 patients. For 3/17 patients the GTV volume increased, in all three cases by more than 10%. The average, standard error and range of the Dice for each of the six structures are shown in Figure 1. For the target ROIs, the DIR-algorithms were more accurate for the GTV than the nodal-GTV. This is expected due to the GTV being more easily defined on a CT due to higher contrast between the GTV and the surrounding lung. For the spinal cord and the lungs, the three algorithms were able to accurately track the clearly defined boundaries of the organs. For the esophagus, where the organ boundaries are not as clear on a CT scan, some discrepancies were observed.

**Table 1:** Changes in GTV volume between pre- and mid-treatment images

**Patient**	**Pre-treatment**	**Mid-treatment**	**% difference**
1	15.8	14.5	8.2
2	150.5	106.2	29.4
3	96.6	111.4	-15.3
4	39.4	39	1.0
5	32.9	26.4	19.8
6	7.9	7.8	1.3
7	55.8	47	15.8
8	59.6	65.7	-10.2
9	158.6	120.9	23.8
10	18.7	17.2	8.0
11	115.7	104.3	9.9
12	17.9	17.3	3.4
13	3.1	2.9	6.5
14	182.1	148.8	18.3
15	46.3	56.9	-22.9
16	10.9	10.3	5.5
17	66.6	65.5	1.7
Average	63.44	56.59	6.1

**Figure 2:**
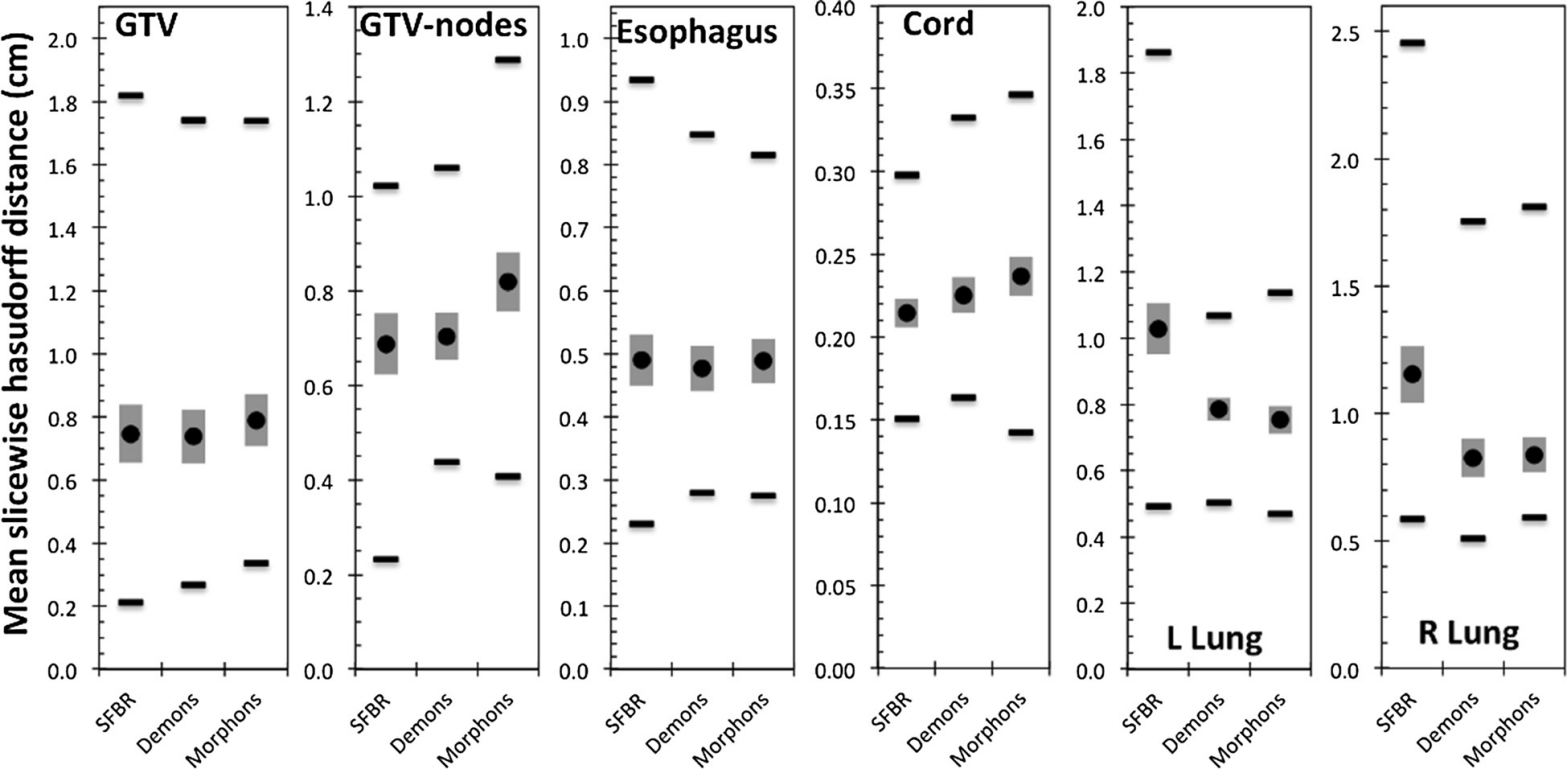
**Mean slice wise Hausdorff distances to agreement for all structures.** The lower the MSHD, the better the agreement between the ROIs. The black circle is the average over all patients, the grey vertical bar is the standard error and the black horizontal lines are the ranges.

**Figure 3:**
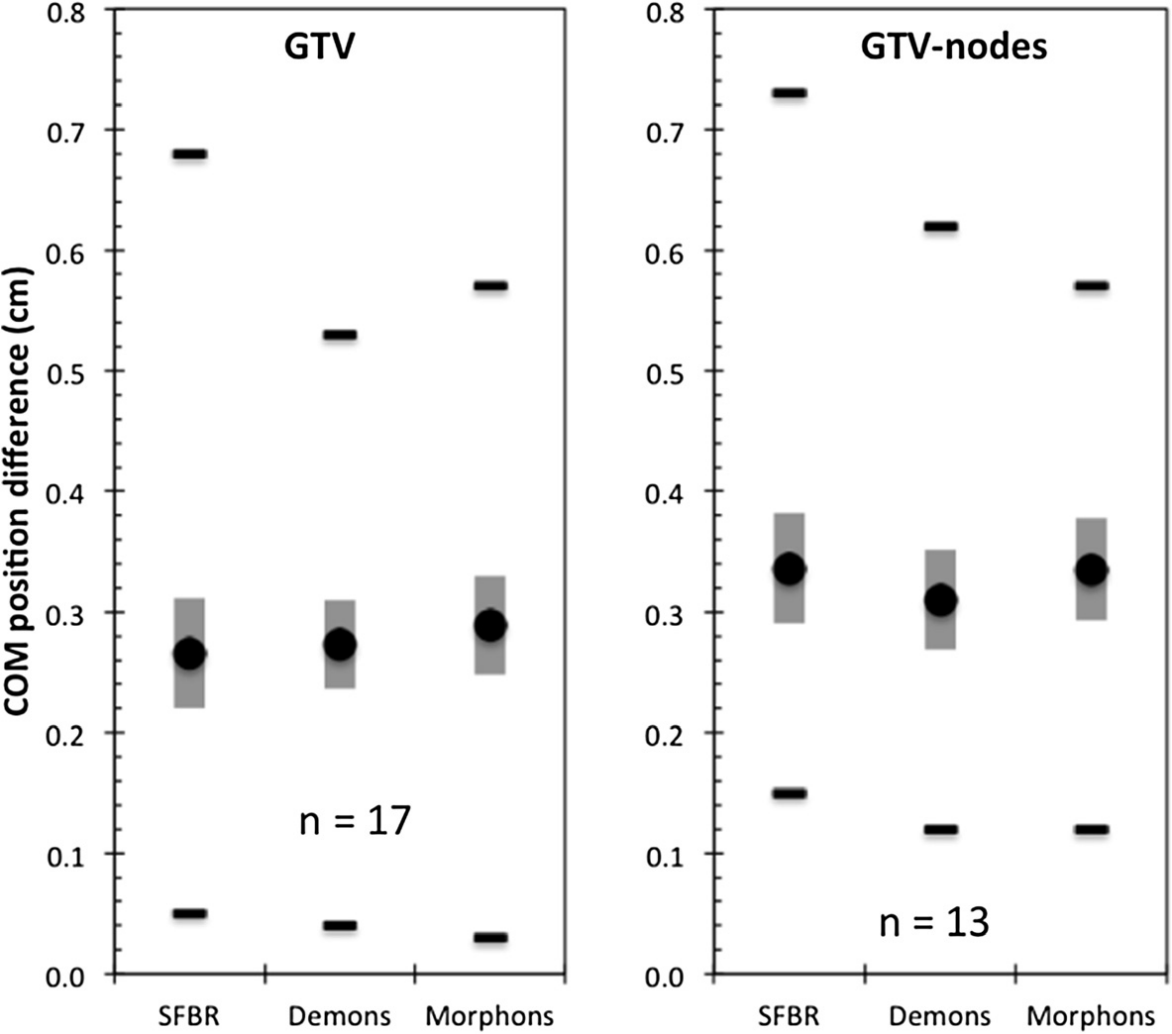
**The COM location difference between the DIR-propagated target ROIs and the physician-drawn ROIs.** The black circle is the average over all patients, the grey vertical bar is the standard error and the black horizontal lines are the ranges.

No statistically significant differences between the three algorithms were observed with the exception of the left lung, for which SFBR-propagated ROIs had a lower average Dice score than those from Demons and Morphons (*p* = 0.0030 & *p* = 0.0001 respectively). Figure 2 shows the MSHDs for the six structures. For the lungs, the SFBR-propagated ROIs had statistically significantly higher MSHDs than those from the Demons and Morphons algorithms (*p* = 0.009 and *p* = 0.005 respectively for left lung, *p* = 0.025 and *p* = 0.027 respectively for right lung). For all other ROIs, there was no statistically significant difference between the algorithms. The average COM differences (Figure 3) for the GTV-tumor and nodal-GTVs ranged from 0.27 – 0.29 cm and 0.31 – 0.37 cm. There was no statistically significant difference between the algorithms for COM location.

**Figure 4:**
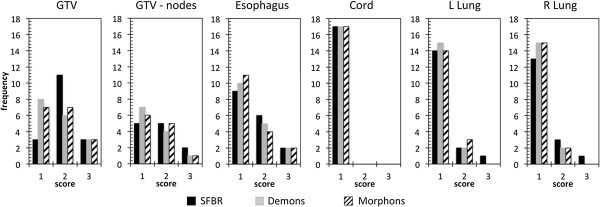
Histograms of the physician scores for each ROI.

**Figure 5:**
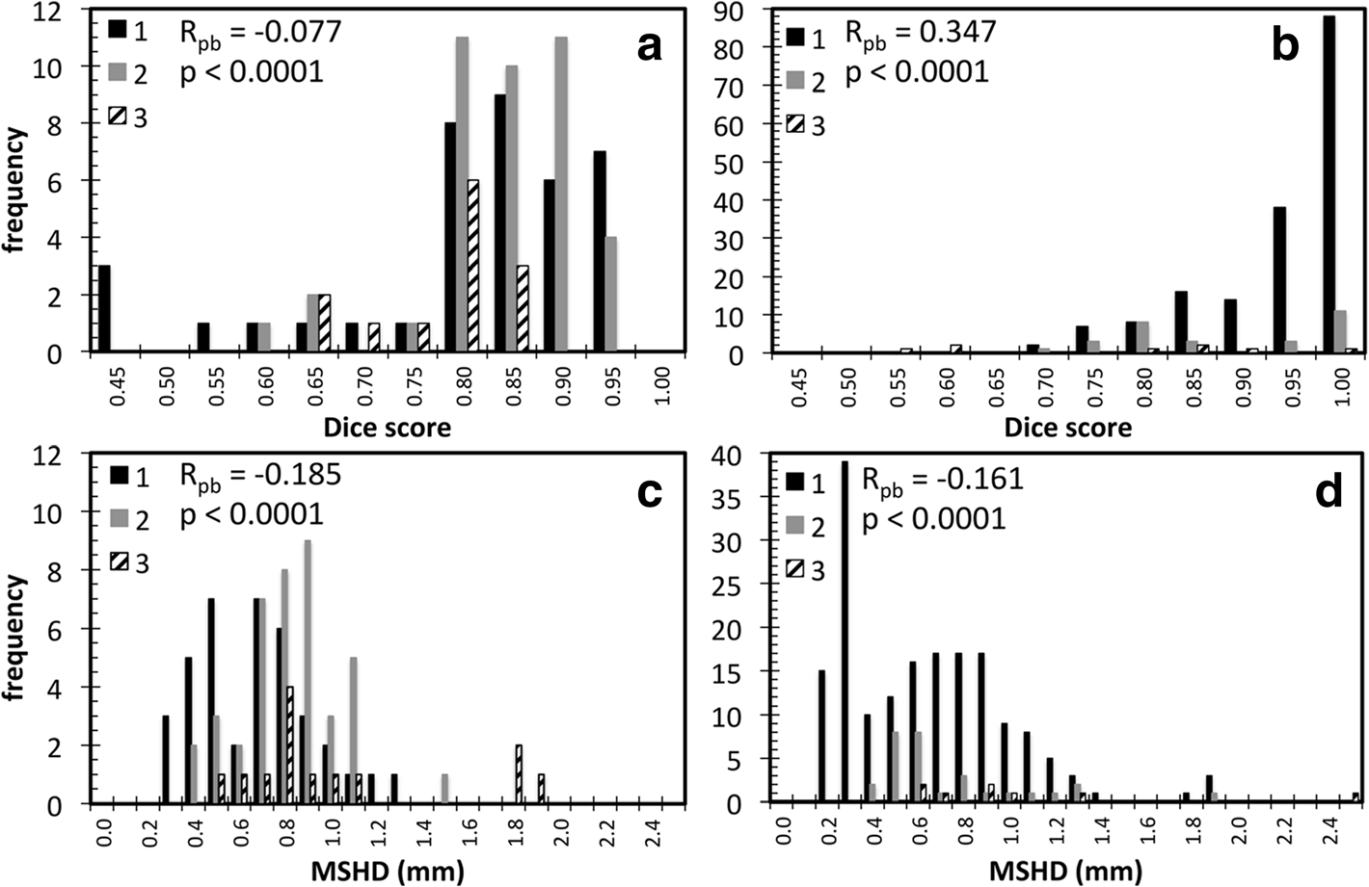
**Histograms of the metric scores grouped into physician scores (a) Target Dice scores (b) OAR Dice scores (c) Target MSHDs and (d) OAR MSHDs.** The Point-biserial correlation test was performed to compare ROIs with a score of 1 (no editing required) with ROIs with a score of 2 or 3 (minor or major editing required). The value of *R* and *p* is given on each chart.

The results of the qualitative evaluation of the ROIs by an expert physician are presented in Figure 4. The majority of the target ROIs were scored either 1 (41%) or 2 (44%), with 15% of targets scored 3. The majority of the OARs were scored 1 (82%) or 2 (14%), with 4% of OARs scored 3, of which 3% were esophagus. All scores of 3 for the esophagus were for the same two patients for all three algorithms. For the cord, all ROIs were scored 1. There was no statistical difference between three algorithms based on the physician scores. The physician scores were compared with the Dice scores and MSHDs to determine any correlation between the two methods of evaluation. Figure 5 shows the histograms of the Dice scores and MSHDs split up between the ROIs scored 1, 2 or 3. The point-biserial test was performed to compare the ROIs scored 1 (no edits required) with the ROIs scored 2 or 3 (minor or major edits required) for the targets and the OARs. A weak negative correlation was observed between the Dice scores and MSHD and physician scores for the targets. For the OARs, moderate positive correlation between the Dice scores and physician scores was observed but weak negative correlation between the MSHDs and physician scores was observed.

## Discussion

This study investigates the ability of three DIR algorithms to automatically propagate ROIs in adaptive lung radiotherapy. The three algorithms used in this study all derive deformations between two images using different properties of the image – image intensity, landmarks and quadrature phase difference with the Demons, SFBR and Morphons algorithms respectively. Despite the differences between the algorithms in terms of how they derive deformation fields, there was very little difference in the quality of automatically propagated ROIs between the three algorithms. As expected, all three algorithms had limited success with tracking anatomical changes in the esophagus and nodal-GTVs, most likely due to limited soft tissue contrast in the kVCT image around these structures. For clearly defined anatomical structures such as the cord, lungs and GTV, the performance of the algorithms was superior to that for low contrast organs. The high Dice scores for the lung need to be taken with some context – the Dice score relies on the volume of the ROIs therefore for very large ROIs such as the lung, a large deviation between two volumes has to occur before the Dice score starts to decrease. Therefore the MSHD and physician scores are likely to be more relevant metrics for the lungs.

**Figure 6:**
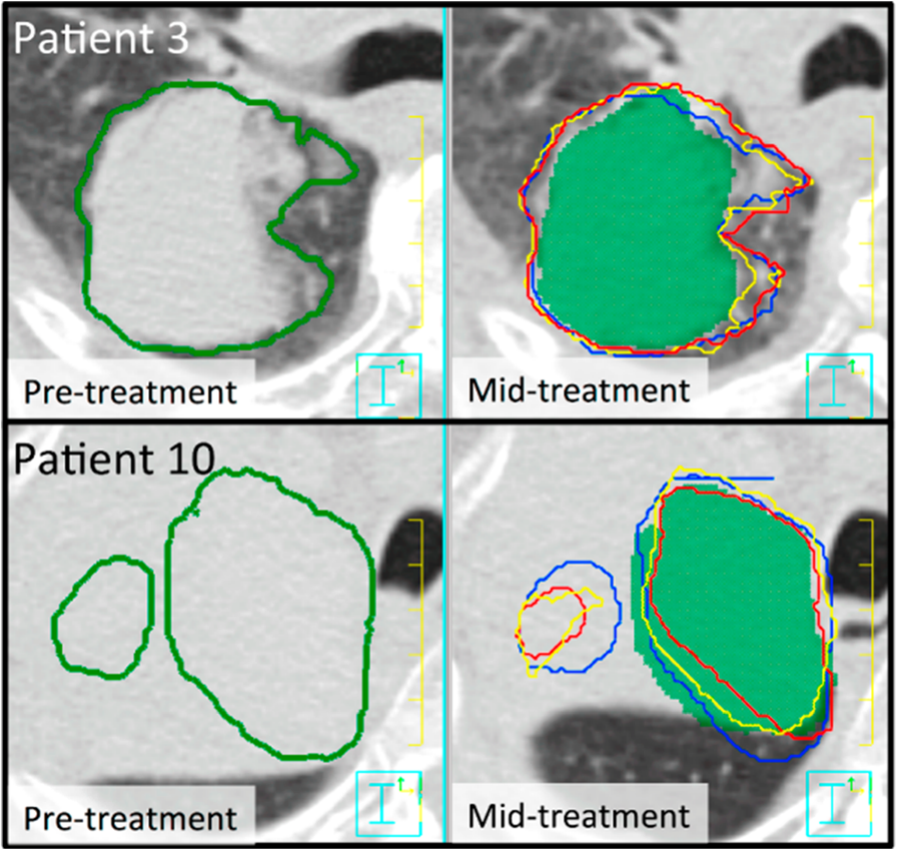
**The left images show the pre-treatment kVCT with the GTV outlined.** The right images show the mid-treatment kVCT image with the physician-drawn GTV (green colourwash), Demons (red), SFBR (blue) and Morphons (yellow) propagated GTVs. Clear differences in the GTV definition between the images are shown for both patients.

Comparing the physician scores for the GTV and the nodal-GTV, the nodal-GTVs received a higher rate of scores of 1 and lower rate of scores of 3 than the GTV. This difference between the GTVs and the nodal-GTVs did not correlate well with the Dice scores; the GTVs had greater average Dice scores than the nodal-GTVs. The range of the MSHDs however was smaller with the nodal-GTVs. These discrepancies could partly be due to the differences in the ROI sizes – small changes in the physically smaller nodal-GTV ROIs would have a larger impact on Dice scores than the same change in the larger GTV ROI. There may also be clinical reasons for the discrepancy. Clinically, the mid-treatment nodal-GTV volumes were delineated on the mid-ventilation phase of a 4DCT scan. Thus, the DIR algorithms had access to the same image information as that used clinically to determine nodal-GTV borders. This suggests that the anatomy surrounding the nodal-GTV could provide adequate information for the algorithms to derive the local nodal-GTV deformation or that there was minimal deformation of the nodal-GTV between the two scans. This finding could also be explained by the fact that visualizing nodes using a non-contrast-enhanced CT is difficult, therefore a propagated contour might be considered adequate, whereas a re-delineation of the node introduces again some intra- or inter-observer variability. It should be noted however that any disagreement between the automated or manually delineated nodal-GTV ROI and the actual nodal-GTV would possibly be different, since the automated method could lead to systematic differences whereas the manual delineation could lead to random differences. A definitive answer to this question is out of the scope of this study but would warrant further investigation. The inconsistent results with the propagated and physician delineated GTVs represented by the Dice scores are in our opinion also partly caused by the observer variations. Another factor contributing to this is the fact that often the target volume definition is modified between the two images, based on information not available in the CT image. An example of this is shown in Figure 6, which shows for two patients the differences in GTV definition between the two images, based on information not contained in the CT image hence not accessible to the DIR algorithms. Thus it is highly recommended that automatically propagated target volumes be thoroughly assessed by the treating physician, since the shape of the target structures in the pre-treatment images influence the shape of the propagated structures independent of the employed algorithm, if there are only weak contrast or gradient differences in the mid-treatment image.

The automatic propagation of the ROIs using the three algorithms took on average 3 min 39 s (Demons), 4 min 16 s (SFBR) and approximately 60 min (Morphons). Although at the time of use, none of the algorithms were optimised for speed, Demons and SFBR both have elements that are multi-threaded and were performed on a 16-core Sun Fire x4450 system. DIR with the Morphons algorithm in contrast was performed using 8 dual-core Intel Xeon X5550 2.66 GHz processors but the implementation was not (yet) multi-threaded. Use of 16 available cores and assuming a fully multi-threaded implementation of the algorithm with no serial component in the code remaining could theoretically could reduce calculation times 16-fold and would result in a calculation time of the same order of magnitude as that for Demons and SFBR.

Although the physician scores represent the opinion of one expert physician, this is still a useful test of the clinical utility of automatically propagated ROIs in adaptive radiotherapy. The weak correlation between the physician scores and the Dice scores and MSHDs, suggest that the use of Dice scores and MSHDs alone are not sufficient for complete evaluation of automatically propagated ROIs in the lung/thorax region. The fact that 30% of structures required editing implies that the treating physician must assess all automatically propagated ROIs to ensure all propagated ROIs are sufficiently accurate for adaptive assessment and re-planning. The comparison of the automatically propagated with the physician drawn ROIs must be done in the context of inter-observer variation. In this study all ROIs were delineated by an expert physician using PET/CT image data, which significantly reduces inter-observer variation, thus it is expected that the physician drawn ROIs used for comparison in this study would not vary significantly from those obtained from multiple observers [27,28].

## Conclusions

Three DIR algorithms were used to automatically propagate both normal tissue and target volumes in repeat lung kVCT scans. Reasonably good agreement with physician drawn contours was observed for normal tissues. DIR-propagated nodal-GTV and esophagus structures were not as accurate, most probably due to less soft tissue contrast for these structures. This system is nevertheless a major step forwards for fast and accurate delineation for adaptive radiotherapy. Further work should include a robust analysis of efficiency gains when using automatic propagation of contours in adaptive radiotherapy.

## Abbreviations

DIR: Deformable image registration; GTV: Gross tumour volume; PTV: Planning target volume; PET: Positron emission tomography; CT: Computed tomography; ROI: Region of interest; MSHD: Mean slicewise hausdorff distance; SFBR: Salient feature based registration; OAR: Organ at risk; kVCT: Kilovoltage computed tomography; MVCT: Megavoltage computed tomography; RTPS: Radiotherapy treatment planning system.

## Competing interests

Author NH performed this work whilst employed at the University of Wisconsin-Madison as a post-doctoral scientist funded through a grant awarded to WAT at UW from Philips Radiation Oncology Systems.

Author WAT received grant funding from Philips Radiation Oncology Systems.

NH & WAT receive royalties from a patent covering one aspect of deformable image registration awarded to NH and WAT (not included in this manuscript) which is managed by Wisconsin Alumni Research Foundation (WARF).

Author KAB is an employee of Philips Radiation Oncology Systems.

## Authors’ contributions

All authors contributed to manuscript preparation. NH & WvE performed the registrations and quantitative analysis of the registration. KB performed programming of the registration algorithms. WAT assisted in study design and interpretation. DdR performed qualitative analysis of the registration results. All authors read and approved the final manuscript.
